# Evaluating Tannins and Flavonoids from Traditionally Used Medicinal Plants with Biofilm Inhibitory Effects against MRGN *E. coli*

**DOI:** 10.3390/molecules27072284

**Published:** 2022-03-31

**Authors:** Niclas Neumann, Miriam Honke, Maria Povydysh, Sebastian Guenther, Christian Schulze

**Affiliations:** 1 Pharmaceutical Biology, Institute of Pharmacy, University of Greifswald, Friedrich-Ludwig-Jahn-Straße 17, 17489 Greifswald, Germany; niclas.neumann@uni-greifswald.de (N.N.); miriam.honke@gmx.de (M.H.); sebastian.guenther@uni-greifswald.de (S.G.); 2 Saint Petersburg Stat Chemical Pharmaceutical Academy, St. Prof. Popova, 14, 197022 St. Petersburg, Russia; mpovydysh@yandex.ru

**Keywords:** tannins and flavonoids, anti-biofilm, *E. coli*, *Epilobium*, *Filipendula*, R. chamaemorus, biological activities, ethnobotany

## Abstract

In the search for alternative treatment options for infections with multi-resistant germs, traditionally used medicinal plants are currently being examined more intensively. In this study, the antimicrobial and anti-biofilm activities of 14 herbal drugs were investigated. Nine of the tested drugs were traditionally used in Europe for treatment of local infections. For comparison, another five drugs monographed in the European Pharmacopoeia were used. Additionally, the total tannin and flavonoid contents of all tested drugs were analyzed. HPLC fingerprints were recorded to obtain further insights into the components of the extracts. The aim of the study was to identify herbal drugs that might be useable for treatment of infectious diseases, even with multidrug resistant *E. coli*, and to correlate the antimicrobial activity with the total content of tannins and flavonoids. The agar diffusion test and anti-biofilm assay were used to evaluate the antimicrobial potential of different extracts from the plants. Colorimetric methods (from European Pharmacopeia) were used for determination of total tannins and flavonoids. The direct antimicrobial activity of most of the tested extracts was low to moderate. The anti-biofilm activity was found to be down to 10 µg mL^−1^ for some extracts. Tannin contents between 2.2% and 10.4% of dry weight and total flavonoid contents between 0.1% and 1.6% were found. Correlation analysis indicates that the antimicrobial and the anti-biofilm activity is significantly (*p* < 0.05) dependent on tannin content, but not on flavonoid content. The data analysis revealed that tannin-rich herbal drugs inhibit pathogens in different ways. Thus, some of the tested herbal drugs might be useable for local infections with multi-resistant biofilm-forming pathogens. For some of the tested drugs, this is the first report about anti-biofilm activity, as well as total tannin and flavonoid content.

## 1. Introduction

The fight against multi-resistant bacteria is of great importance. Due to the increasing resistance of bacteria to antibiotics, it is becoming increasingly difficult to treat trivial infections. MRGN *E. coli* strains, which are also classified as particularly critical by the WHO, play a special role here [1,2]. In the pre-antibiotic era, medicinal plants were frequently used to treat local infections. With the development of modern antibiotics, their use steadily declined due to the supposedly better efficacy of antibiotics. However, since multi-resistant pathogens are on the rise due to the ubiquitous usage of antibiotics, and the development of new antibiotics is nearly zero, some new therapy approaches have been suggested. One of them is the idea not to attack the bacteria itself, but to reduce their virulence factors [3]. The thusly weakened bacteria can then be fought more effectively by the patient’s immune system. In this therapy approach, the development of new resistances should be minimized due to reduced forced evolution. One of the targeted main virulence factors is the biofilm, which can be produced by many bacteria. A biofilm protects the bacteria against external influences and xenobiotics like antibiotics. It also provides an environment for quorum sensing and exchange of resistance factors. Therefore, some of the main survival and evolutionary successes of multi-resistant bacteria might be due to the capability of biofilm formation [4]. Tannin- and flavonoid-rich plants and drugs have been proven in the past to affect biofilms of bacteria [5,6,7]. On the other hand, it is known that some plant polyphenols, especially tannins, directly inhibit bacteria by denaturation of bacterial proteins [8,9]. As this is a physical effect, it is highly unlikely that the bacteria develop any resistance to tannins. Due to this dual inhibitory effects against bacteria, tannin- and flavonoid-rich plants are in scientific focus again. In the course of ethnopharmacological studies, many medicinal plants are rediscovered, the constituents of which show promising activities. Therefore, the aim of this study was to evaluate extracts from traditionally used medicinal plants, known for their anti-infective activity, for their biofilm inhibitory and general antimicrobial properties. For this reason, particular attention was paid to plants with high content of tanning agents that are yet unknown to the European Pharmacopoeia (Ph.Eur.) but are ethnopharmacologically used for infections. For comparison, plants with well-established use and monographs in the Ph.Eur. were used. These plants were *Hamamelis virginiana* (leaves), *Arctostaphylos uva-ursi* (leaves), *Quercus* sp. (bark) and *Cinchona* sp. (bark). As Europe has a long tradition of herbal medicine, traditional plants that are used against infections were tested for their activity and content of tannins and flavonoids. Some of these plants are also monographed in the Ph.Eur., but often used for other indications. Samples were selected by the Department of Pharmacy, University of St. Petersburg, with a choice of so-used traditional anti-infective drugs known for their tanning agent content. The selected plants were *Aronia melanocarpa*, *Potentilla palustris*, *Epilobium angustifolium*, *Geum rivale*, *Filipendula ulmaria*, *Persicaria bistorta*, *Rubus chamaemorus*, and *Sanguisorba officinalis*.

*Aronia melanocarpa* is known for its high concentration of antioxidants and Vitamin C. The fruits are used as food or as a nutritional supplement. They are also known for their content of tanning agents, which can be recognized by the adstringent taste of the fruits. Aronia has been increasingly promoted as a “super food” with various health promises for several years. Antioxidant and vascular strengthening effects are attributed to it, as well as—especially in alternative medicine circles—cancer-inhibiting properties. In the literature, positive evidence for these effects can be found, but a definitive proof is missing until now [10,11]. A biofilm-inhibiting effect on *Bacillus cereus* has been demonstrated for chokeberry fruit compounds [12]. *Potentilla palustris* is closely related to the species *Potentilla erecta* and *Potentilla anserina,* which are both monographed in the European Pharmacopoeia. Both are used for infections of the oral cavity and spasmodic complaints of the lower gastrointestinal tract. *Potentilla palustris* itself is not monographed in the Ph.Eur., but has a similar spectrum of components [13]. For another close relative, *Potentilla visianii*, biofilm-inhibiting activity was reported [14], thus implicating a high potential for further investigations for *P. palustris*. *Epilobium angustifolium* is a very common plant in Central Europe which can be found near ruderal posts and embankments. It is not very commonly used as a medicinal plant. However, the EMA published a monography in the year 2016 for use in benign prostatic hyperplasia. In Russia, the fermented leaves were used as a black tea substitute, but they do not content caffeine. *Geum rivale* was reported to have been traditionally used against minor skin infections and gastrointestinal tract disease, and some anti-arthritic usages were described [15,16]. The literature reports significant amounts of phenolic acids, tannins, and flavonoids. The main component of the phenolic acid fraction consists largely of ellagic acid. For ellagic acid, there is evidence in the literature of a biofilm-inhibiting effect [17,18]. Recently, two more flavonol bis-3,7-glucuronides were discovered and their structure elucidated [19]. *Filipendula ulmaria* has a long tradition in Central Europe as it contains large amounts of salicylic acid derivatives, which have been long-known for their pain relieving and antipyretic properties. Meadowsweet herb is monographed in Ph.Eur. whereas the quality descriptions mainly refer to the content of methyl salicylate or essential oil. The European Scientific Cooperative on Phytotherapy attributes meadowsweet a supportive effect in banal colds [20]. The content of tannins in meadowsweet are known, but so far no sufficient studies have been made regarding specific effects of these components. *Persicaria bistorta* belongs to the Polygonaceae, a family known for its high content of tannins. There is also a monograph in the European Pharmacopoeia. The rhizome of the plant was used traditionally for diarrhea and pharyngitis. *Rubus chamaemorus* is not known to the German or European Pharmacopoea. The fruits of *R. chamaemorus* are used in Scandinavia as jams or as flavoring for beverages. They contain a high amount of benzylic acid. The leaves were used against diarrhea and minor infections. The plant itself grows slowly as typical bog flora. *R. chamaemorus* is endangered because of the steady decline of bog land, and due to its slow growth and poor dissemination via seeds. *Sanguisorba officinalis* rhizome and root have been described in diarrhea and dysmenorrhea by traditional medicine. Nowadays, its use is not very common. However, in the literature, biofilm-inhibiting effects were described in *Staphylococcus aureus* [21]. Overall, all of the plants described above are known to have some kind of tanning agent as their main component. Some of them had been tested before for biofilm inhibition. However, the general antimicrobial effects were frequently known to the literature but are rarely systematically investigated against highly resistant strains.

The aim of this study was to screen and identify herbal drugs with antibacterial and biofilm-inhibiting activity. The study focused on problematic bacteria strains including MRGN *E. coli* and a yeast. A second objective of this study was to evaluate the total tannin and flavonoid yield of the tested herbal drugs. Finally, correlation analyses were performed to check for possible correlations between tannin or flavonoid contents and biological activity of the investigated drugs.

## 2. Results and Discussion

### 2.1. Tannin and Flavonoid Content

Table 1 summarizes the tannin and flavonoid content of the tested drugs. The tannin and flavonoid content was determined separately with the total biomass using the methods described. The dried and pulverized material was used for the assay in each case. Tannin contents between 2.15% (*Cinchona* bark) and 10.41% (*Epilobium* leaves) were found. Interestingly, the highest contents were found in drugs that are usually not regarded as typical tannin-rich drugs (e.g., *Epilobium*, *Rubus*, *Sanguisorba* and *Filipendula*). On the other hand, it has to be kept in mind that the tannins were determined with a Ph.Eur. standardized method. The tannin content was normalized to the redox potential of pyrogallol, suggesting that there might exist biases due to different redox potentials of the different tannins (e.g., catechines vs. gallotannins) [22].

The total flavonoid content ranged from 0.09% (*Aronia* fruit) to 1.92% (*Epilobium* herb). Although these values appear quite low, they cover the range typically found in drugs used because of their flavonoid content (e.g., Elder flower Ph.Eur. ≥ 0.8%, Birch leaf Ph.Eur. ≥ 1.5%) and analyzed with Ph.Eur. methods. For most plants (12 out of 14), the boric acid-oxalic acid method showed the higher values for flavonoid content. The only exceptions were *E. angustifolium* (both values equal) and *H. virginiana* (higher value for the aluminum chelate method). Concerning the mechanism of the two analysis methods, this fits well with the values found. The aluminum chelate method only records those flavonoids that can be hydrolyzed with acetone-hydrochloric acid. Only their aglycons can be shaken out with ethyl acetate after hydrolysis and then react with the aluminum chloride. This mainly affects *O*-glycosides. The boric acid-oxalic acid method, on the other hand, captures more flavonoids. Any flavonoid that can be extracted with hot 60% ethanol and contains a free vinylogous carboxylic acid or hydroxy group can, in principle, react. In addition to most *O*-glycosides, this also applies to many *C*-glycosides and all aglycons. Since more compounds are recorded overall, the values for the boric acid-oxalic acid method must be higher. Only for *Hamamelis* leaves this does not apply. Many flavonoids seem to be contained here, which are only easily accessible for analysis after hydrolysis.

### 2.2. Antimicrobial Activity

The data obtained in the biological tests are summarized in Table 2 and Table 3.

#### 2.2.1. Agar Diffusion Test

The general antimicrobial activity of the drugs was evaluated with 1 mg of the obtained extracts, tested in the agar diffusion test with seven test pathogens (two gram positive, four gram negative bacteria, and one yeast). The solvent controls showed no inhibition, the positive control revealed the following inhibition diameter (mean ± SD, *n* = 7): 24 ± 1.4 mm (*S. aureus*), 38 ± 1.7 mm (*B. subtilis*), 27 ± 3.5 mm (*E. coli*), 20 ± 0.4 mm (*E. coli* PBio 729), 17 ± 0.7 mm (*E. coli* PBio 730), 29 ± 2.3 mm (*P. aeruginosa*), and 25 ± 1.5 mm (*C. maltosa*). The DCM extracts, containing the non-polar compounds, showed only little activity. Mentionable activity can be found for *K. lappacea* root extract, which inhibited four of the five tested bacteria. Except for *A. melanocarpa* fruit extracts, all of the tested methanolic extracts showed activity against at least two pathogens. Candida was most sensitive towards the extracts: 11 of the 14 showed activity. With an inhibition zone of 25 mm in diameter, *E. angustifolium* leaf extract was most potent against the yeast. The methanolic extracts are typically rich in medium-polar compounds like tannins and flavonoids. The good activity of tannins and flavonoids is well known [23,24] and supports the results. The antimicrobial activity of the water extracts was lower compared to the methanol extracts. Moderate activity can only be observed for *Candida*, which may also be due to the presence of polar tannins and flavonoids in the water extracts. In general, the antimicrobial potential of the extracts is low, especially considering the relatively high concentrations of the extracts (1 mg). Further, the inhibition diameters are only low compared to the positive controls. The observed antimicrobial effects where more prominent on gram-positive organisms. *S. aureus* was the most sensitive organism, and effects with most of the methanolic extracts could be observed. Furthermore, some of the dichloromethane and water extracts showed activity, too. Another exception was *C. maltosa*, which was also sensitive to most methanolic extracts and many water extracts. However, since the direct antimicrobial activity was not the main focus of this study, no MIC values were determined. The generally weak to moderate antimicrobial activity of the extracts indicates that direct pathogen inhibition might not be the only reason for the successful traditional use of the plants for infections. Hence, the question arose whether the main effects may be induced by tannins which target the virulence factors, like the biofilm, instead of a direct antimicrobial effect [25]. This thus lead to the conduction of an anti-biofilm assay with gram-negative test organisms to prove the hypothesis.

#### 2.2.2. Anti-Biofilm Activity

Biofilm inhibitory effects were tested with two *E. coli* strains (PBio 729 and PBio 730 [26], see material and method section). Curly (a protein) and cellulose (a polysaccharide) production, which is associated with biofilm formation, was evaluated by dying the structures. As tannins especially affect proteins, the influence of the extracts on curly production was the most interesting aspect for this study. Due to their high curly production, these *E. coli* strains were selected for testing.

None of the tested DCM extracts showed any biofilm inhibition. Nine of the 14 tested water extracts showed weak anti-biofilm activity (MAC 100–200 µg mL^−1^). The most active were the methanolic extracts. Except for *Aronia*, *Cinchona*, and *Krameria*, all of the tested methanol extracts showed biofilm inhibitory effects for both of the tested bacterial strains. *Aronia* showed to have no activity against the tested *E. coli* strains at all. *Cinchona* and *Krameria* showed weak activity, but not on both strains. The activity (MAC 200 µg mL^−1^) of *Cinchona* could be observed for *E. coli* PBio 730. For *Krameria* the same activity could be observed for *E. coli* PBio 729. *P. bistorta* extract was most active; the MAC was found to be 10 µg mL^−1^ for both of the tested bacteria. Good biofilm inhibition has also been observed for *F. ulmaria*, (MAC 30 µg mL^−1^), *S. officinale* (MAC 20 and 40 µg mL^−1^), and *A. uva-ursi*, *H. virginiana*, and *R. chamaemorus* (MAC each 20 and 50 µg mL^−1^). In general, *E. coli* PBio 730 was more sensitive to the extracts. The MAC is lower for eight of the 13 extracts, compared to *E. coli* PBio 730. Altogether, many of the traditionally used medicinal plants showed at least equal biofilm inhibitory effects as the commonly used drugs (e.g., oak bark, hamamelis leaves). Some of the methanol extracts were even more active than the positive control EGCG (MAC 35 µg mL^−1^), and this was a first indication that tannins and flavonoids might be responsible for the activity. As these natural compounds typically contain polar hydroxy groups, it was therefore to be expected that tannins and flavonoids would accumulate in the methanol extract [27,28] rather than in the DCM extracts. Since the extraction was performed stepwise, a low activity for the water extracts is plausible as the suspected active compounds were already extracted with methanol. Since tannins and flavonoids are also known for antimicrobial activity [5,6,7], a correlation analysis between tannins, flavonoids, and biological activity was considered reasonable.

### 2.3. Correlation Analysis

**Figure 1:**
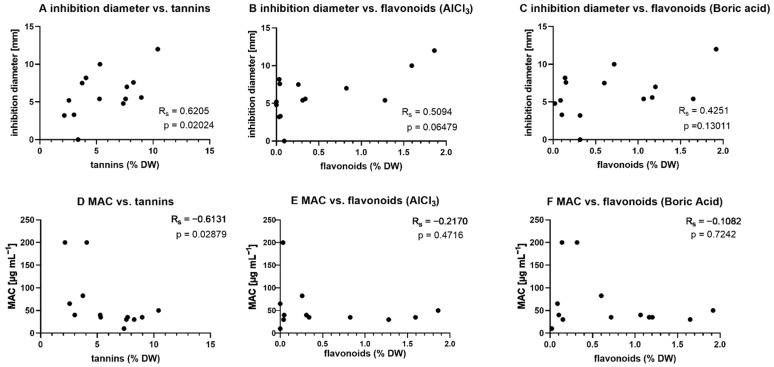
Correlation of tannin and flavonoid content with biological activity for methanol extracts. Statistical analysis: Spearman Rank Correlation Test (GraphPad Prism) [32]. Correlation is assumed if correlation coefficient is significantly (*p* ≤ 0.05) non-zero (R_s_ > ±0.10) [33].

To further investigate whether the biological effects of the extracts might be due to the flavonoid or tannin content, a Spearman correlation analysis was performed. For correlation analysis, the antimicrobial and anti-biofilm activity of the methanol extracts was used. On one hand, these extracts were most active, but on the other hand, the investigated tannins and flavonoids are medium polar to polar and therefore enriched in the methanol extracts. A summary of the statistical data can be found in Table S1. Figure 1A–C displays the correlation between tannins or flavonoids and the mean inhibition diameter of the methanol extracts. The Spearman coefficient (R_s_) indicates that there is a moderate (R_s_ = 0.6205) and significant (*p* = 0.02024) correlation between tannin yield and activity [29]. The correlation is positive, meaning that with increasing tannin yield, the mean inhibitory activity increases. No correlation can be observed when comparing flavonoid yield and biological activity. It made no difference whether the flavonoids were quantified using the aluminum chelate or the boric acid-oxalic acid method. Although the Spearman coefficient (R_s_ 0.5094 and 0.4251, respectively) indicated a possible correlation, this was not significant. When analyzing the correlation between anti-biofilm activity and tannin or flavonoid content, negative Spearman coefficients could be observed. This indicates that with increasing yield of tannins or flavonoids, the MAC decreases, thus the anti-biofilm activity increases. However, only for tannins, there is a moderate (R_s_ = −0.6131) and significant (*p* = 0.02879) correlation. For flavonoids, no statistical significant correlation can be observed. This is somewhat remarkable, as flavonoids are commonly regarded as active biofilm-inhibiting substances [5,6,7,30]. There are two approaches to explain how the data correlation might have been biased resulting in differences from the literature data. First, it is possible that the photometric quantification method does not fully capture the flavonoids relevant for biofilm inhibition. Since the content is always given as “calculated as hyperoside”, this seems plausible because hyperoside has only comparatively low anti-biofilm activity [31]. Second, it is possible that the assay used to evaluate biofilm inhibition does not reflect the full potential of the flavonoids. The assay used is based on the staining of two matrix components of the biofilm, but a biofilm is much more complex and can also be influenced elsewhere.

**Figure 2:**
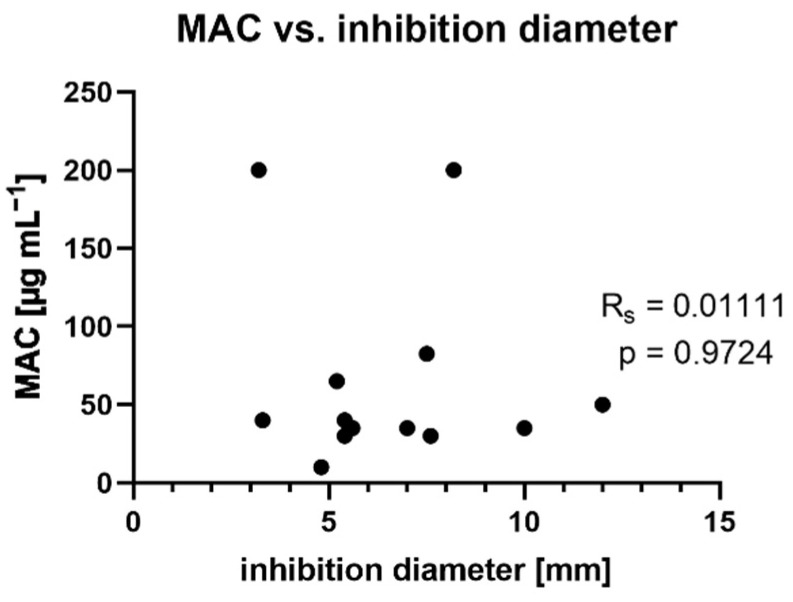
Correlation of anti-biofilm and antimicrobial activity for methanol extracts. Statistical analysis: Spearman Rank Correlation Test (GraphPad prism) [32]. Correlation is assumed if correlation coefficient is significantly (*p* ≤ 0.05) non-zero (R_s_ > ±0.10) [33].

In Figure 2 it is shown that there is no statistical correlation between inhibition diameter and MAC, thus indicating no coherence of antimicrobial activity and anti-biofilm activity. Tannins showed both direct antimicrobial and anti-biofilm activity. Since the two effects are not related, it can be concluded that the tanning agents inhibit microorganisms in different ways. On one hand, the pathogens are attacked directly, however on the other hand their virulence (in the form of biofilms) is influenced. This makes tannins interesting as potential therapeutic agents for infections, respectively underlining the well-established use of these natural compounds with these indications. However, since the effects are at least partly of a physical nature (denaturation of proteins), only direct, i.e., local, treatment options can be considered. On the other hand, the development of bacterial resistance to physical effects is hardly possible.

### 2.4. HPLC Fingerprint Analysis

**Figure 3:**
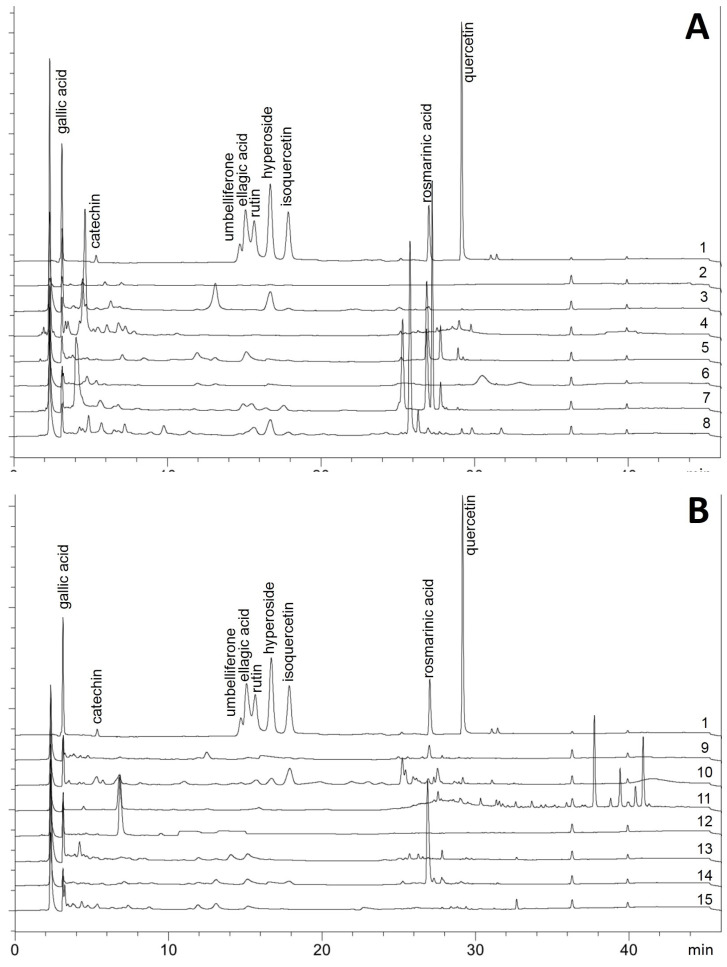
HPLC fingerprints of the investigated plant extracts at 254 nm (MeOH, each 1 mg mL^−1^). (**A**) 1 reference mixture (0.1 mg mL^−1^); 2 *Aronia melanocarpa* (fruits), 3 *Arctostaphylos uva-ursi* (leaves), 4 *Cinchona* sp. (bark), 5 *Comarum palustre* (herb), 6 *Comarum palustre* (rhizome), 7 *Epilobium angustifolium* (leaves), 8 *Filipendula ulmaria* (herb). (**B**) 1 reference mixture (0.1 mg mL^−1^), 9 *Geum rivale* (herb), 10 *Hamamelis virginiana* (leaves), 11 *Krameria lappacea* (root), 12 *Persicaria bistorta* (rhizome), 13 *Quercus* sp. (bark), 14 *Rubus chamaemorus* (leaves), 15 *Sanguisorba officinale* (root and rhizome).

Figure 3 shows the HPLC fingerprints of the plant extracts. The separation of the extracts into individual components can be well assessed using the reference substances. After the injection peak at RT (retention time) about 2 min, the small and relatively polar compounds gallic acid (RT 3.5 min) and catechin (RT 5.5 min) are eluted first. Due to the initially very slow increase in elution power up to t 15 min, a range follows in which the moderately polar flavonoid glycosides and phenylpropanoids are separated well. Ellagic acid (RT 13.1 min), rutin (RT 13.8 min), hyperoside (RT 17 min), and isoquercetin (RT 18 min) can be seen. This is followed by rosmarinic acid (RT 27.2 min), and quercetin (RT 29.5 min), which is relatively non-polar due to the lack of sugar in the molecule. In the subsequent wash-out phase (up to t 42.6 min), further, particularly non-polar components can be eluted. If looking at the fingerprints of the extracts, it is noticeable that the contents determined in the colorimetric determinations can be easily explained. If the total flavonoid content is relatively high, there are many and comparatively large peaks in the area of flavonoid glycosides. This appears very clearly, for example, in the extracts from *Arctostaphylos* (3), *Epilobium* (7), *Filipendula* (8), or *Hamamelis* (10). Overall, however, HPLC also confirms that the flavonoids represent only a relatively small proportion of the extract. This can be seen from the overall relatively small peaks compared to the peaks of the standards. The analysis of tanning agents using HPLC is generally difficult because tanning agents are difficult to separate with C18 columns due to their relatively high molecular mass. In addition, some of them are quite polar and are therefore already eluted with the injection peak. Nevertheless, some conclusions can be drawn from the chromatograms. Many of the plants examined, which showed a high content in the total tannin determination, show a conspicuously large number of peaks in the area of rosmarinic acid (RT 25–30 min). This concerns e.g., *Comarum* (herb, 5), *Epilobium* (7), *Filipendula* (8), and *Rubus* (14). However, there are also exceptions: *Arctostaphylos* (3), *Persicaria* (12), and *Sanguisorba* (15) also showed high levels of tanning agents using the colorimetric method, but hardly any peaks in the rosmarinic acid range. Instead, there are more numerous and larger peaks in the area of the reference gallic acid (around RT 2.5 min) and catechin (around RT 5 min). This could indicate that in these plants the gallotannin and catechin tannins predominate, while the other plants contain more tanning caffeic acid esters. All tannin groups appear to be present in *Hamamelis* (10), as peaks appear both in the rosmarinic acid region and between RT 2.5 and 6 min. It is also striking that many very non-polar components (RT 37–42 min) appear in *Krameria* (11). This could be the lignans (“ratanhiaphenols”) typically contained in this root. In the case of *Chinchona* (4), a very large peak appears in the area of the catechins (RT 4 min), but the tannin content is very low. This large peak could be the cinchona alkaloids. Due to the acidic mobile phase, the alkaloids are charged and therefore polar, which explains the early elution. In addition, these substances absorb very well due to their chromophore system, which explains the peak size. Overall, it was possible to use the HPLC method to obtain an approximate overview of the components contained in the methanol extracts. Many peaks can be assigned to substance groups, thus confirming the results of the colorimetric methods.

## 3. Materials and Methods

### 3.1. Plant Material

The following plant material was collected and dried by A. A. Orlova on 13 August 2019 in Lemblovo at the collection nursery of medicinal plants of Saint-Petersburg State Chemical Pharmaceutical University of the Russian Ministry of Health (SPCPU) (Leningrad region, Lembolovo village) [voucher specimen number]:

*Aronia melanocarpa* (fruit) [SPCPU1], *Comarum palustre* (herb and rhizome) [SPCPU2], *Epilobium angustifolium* (leaves) [SPCPU3], *Filipendula ulmaria* (herb) [SPCPU5], *Geum rivale* (herb) [SPCPU4], *Persicaria bistorta* (rhizome) [SPCPU6], *Rubus chamaemorus* (leaves) [SPCPU7], and *Sanguisorba officinalis* (root plus rhizome) [SPCPU8]. Plant samples were identified using the “Illustrated guide to plants of the Karelian Isthmus” [34] and the authentication was confirmed by Professor G.P. Yakovlev (SPCPU, Department of Pharmacognosy). To identify voucher samples, morphological analysis was used to determine (if necessary) the morphometric characteristics of leaves and flowers. Confirmation of the species affiliation of the samples was carried out using international official botanical Internet resources. To search for nomenclatural citations and correct Latin names of species, we used the data of the Internet resource “International Plant Names Index”—IPNI (https://www.ipni.org/, accessed on 21 February 2022). Comparison of voucher samples was carried out with published descriptions of type species, as well as with photographs of type samples of these species posted on the website of the Royal Botanic Gardens, Kew—“Plants of the World online”—POWO (https://powo.science.kew.org/, accessed on 16 August 2019) (see Table S2). The voucher specimens were deposited in the Herbarium of Saint-Petersburg State Chemical Pharmaceutical University of the Russian Ministry of Health (SPCPU).

The plants were chosen because of their ethno-medicinal use in Europe against various infectious diseases. For comparison, some medicinal plants with monographs in the European Pharmacopoeia (Ph.Eur.) with known antimicrobial effects and high tannin and flavonoid contents were chosen and purchased in pharmacopoeia quality from A. Galke GmbH, Bad Grund, Germany: *Arctostaphylos uva-ursi* (leaves) Art.Nr. 132,002, *Chinchona* sp. (bark) Art.Nr. 32,202, *Hamamelis virginiana* (leaves) Art.Nr. 60,002, *Krameria lappacea* (root) Art.Nr. 101,802, and *Quercus* sp. (bark) Art.Nr. 100,602. Dried drugs were pulverized with a drug mill directly before use.

### 3.2. Extraction

To approximately 10 g of the pulverized drugs, 200 mL dichloromethane (DCM) was added. The mixture was stirred (Heidolph magnetic stirrer) for 24 h at 20 °C and filtered. The residue was extracted another two times with each 200 mL of DCM. The collected filtrates were pooled and evaporated to dryness with a vacuum rotation evaporator at 40 °C (Büchi Rotavapor). The whole procedure was repeated with the same biomass with methanol (MeOH), and afterwards, with deionized water (water). Water extracts were reduced to a volume of approx. 20 mL and freeze-dried. The dried extracts were stored at −20 °C until analysis.

### 3.3. Determination of Tannins

All drugs were analyzed with the test “Tannins in herbal drugs” (Ph.Eur. 10.0, monograph 2.8.14), following the prescribed procedure [35]. In short, approx. 500 mg of the pulverized drugs were extracted with boiling water. After filtration, the water extract was divided into two parts. To one part, phosphomolybdotungstic reagent R was added. After pH adjustment with sodium carbonate solution and 30 min reaction time, absorbance at 760 nm was measured (=total polyphenol content). The second part of the extract was shaken with hide powder CRS and filtered. To the filtrate, phosphomolybdotungstic reagent R was added, and after pH adjustment and 30 min of reaction time, absorbance was measured (=polyphenols not adsorbed by hide powder). As standard substance, pyrogallol was used after reaction with phosphomolybdotungstic reagent R. Polyphenols adsorbed by hide powder (=tannin content) were calculated by subtracting the unabsorbed polyphenols from the total polyphenols, each referred to the absorption of the standard substance. A twofold measurement was considered sufficient, since the pharmacopoeia methods are designed and validated to provide correct values already with a single measurement.

The tannin content was calculated on the dried drug. Loss on drying was determined by drying 1.0 g in an oven at 105 °C to mass constancy (data not shown).

### 3.4. Determination of Flavonoids

The Ph.Eur. provides two different methods for determination of total flavonoids. The first method is mostly used for drugs rich in *O*-glycosylated flavonoids. The assay prescription of the Ph.Eur. monograph “Betulae folium” (Ph.Eur. 9.7, 01/2017:1174) was used [36]. In short, flavonoids were extracted and hydrolyzed with a mixture of acetone and hydrochloric acid. Flavonoid aglycons were extracted with ethyl acetate in a separating funnel. The ethyl acetate layer was washed with water. A defined quantity of the extract was added to a solution of aluminum chloride and glacial acetic acid. Absorbance was measured at 425 nm compared to a compensation liquid without aluminum chloride. The total flavonoid yield was calculated and expressed as hyperoside.

For comparison, as for some plants no data were accessible in the literature to the type of flavonoids, the boric acid oxalic acid method according to the Ph.Eur. monograph “Crataegi folium cum flore” (Ph.Eur. 9.7, 01/2010:1432) was also used, as the method is more suitable for *C*-glycosylated flavonoids [36]. The flavonoids were extracted with ethanol (60% *v*/*v*). After evaporation of a defined quantity, the residue was dissolved in methanol/glacial acetic acid and added to solution containing boric acid and oxalic acid. The absorbance was measured at 410 nm against a compensation liquid, and the percentage of total flavonoids was calculated and expressed as hyperoside. A twofold measurement was considered sufficient for both methods, since the pharmacopoeia methods are designed and validated to provide correct values already with a single measurement.

The flavonoid content was calculated on the dried drug. Loss on drying was determined by drying 1.0 g in an oven at 105 °C to mass constancy (data not shown).

### 3.5. Agar Diffusion Test

All extracts were tested against the following pathogens: *Bacillus subtilis* ATCC 6059, *Staphylococcus aureus* ATCC 6538, *Escherichia coli* ATCC 11229, *Escherichia coli* PBio 729, *Escherichia coli* PBio 730, *Pseudomonas aeruginosa* ATCC 27853, and *Candida maltosa* SBUG 700. Test discs (diameter 6 mm) were impregnated with 1 mg of each extract by transferring 50 µL of solutions of 20 mg mL^−1^ extract in the solvent used for extraction onto the test discs. The test discs were maintained for 24 h to allow solvents to evaporate. As solvent control, test discs were impregnated with 50 µL of each solvent and treated as the discs with extracts. An inoculum of the pathogens was suspended in warm Mueller-Hinton-II-Agar and 20 mL of the agar was dispensed in petri dishes. After cooling, the impregnated test disks were applied on the agar. After 24 h incubation at 37 °C, the test discs were removed. For better visualization of the inhibition zones, 5 mL of a 1% (m/V in ethanol 50% *v*/*v*) iodonitrotetrazolium chloride (INT) solution was dispensed on the agar. After 30 min, the remaining INT solution was discarded and the diameter of the inhibition zones were measured. Ampicillin (8 µg, 10 µg, and 50 µg for *S. aureus*, *B. subtilis* and *E. coli* ATCC 11229, respectively), Chloramphenicol (40 µg, *E. coli* PBio 729 and 730), Gentamicin (10 µg, *P. aeruginosa*), and Nystatin (50 µg, *C. maltosa*) were used as positive control.

### 3.6. Biofilm Assay

**Figure 4:**
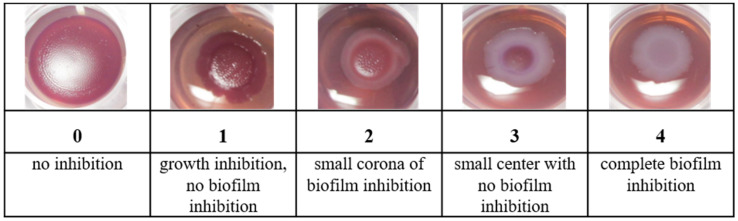
Scheme for the biofilm inhibition score. 0–4: degree of inhibition.

The biofilm inhibitory effect of the plant extracts was evaluated with two biofilm forming *E. coli* strains (PBIO 729 and PBIO 730, previously described [26] as “17433” and “16316”). Both strains produce cellulose and curly in 48 h long-time colonies. Curly and cellulose were used as marker substances for biofilm formation by dying the structures with congo red (cellulose) and coomassie brilliant blue (curly). The anti-biofilm assay was carried out in 24-well-plates as follows (*n* = 2): On the first day, the wells of 24-well-plates were filled with each 200 µg of the extracts (20 µL of a solution of 10 mg mL^−1^ of the extract, dissolved in the extraction solvent). 50 µg of epigallocatechin gallate (EGCG) was used as positive control. On the same day, the test bacteria were taken from frozen (−80 °C) glycerol stocks and streaked out on blood agar plates. The plates were incubated at 37 °C for 24 h. On the second day, one bacteria colony from the blood agar plates was suspended in 4 mL growth bullion and cultivated under constant shaking until an optical density (OD_600_) of 0.5 was achieved. Each well of the previously prepared 24-well-plates was filled with 1.0 mL of congo red agar [37]. Afterwards, 5 µL of the bacteria suspension was positioned in the middle of each well as one single drop. The plate was then incubated at 30 °C for 48 h. The biofilm formation of the colonies was evaluated due to the following score (see Figure 4): 0 (no inhibition), 1 (growth inhibition but no biofilm inhibition), 2 (small corona with no curly/cellulose), 3 (small center with curly/cellulose), and 4 (no curly/cellulose). Biofilm inhibitory effects were defined if at least score “2” was observed. For all extracts with inhibitory effects, the minimal active concentration (MAC) was evaluated by testing extract concentrations from 200 µg mL^−1^ to 5 µg mL^−1^ (*n* = 2) with the test system, as described above.

### 3.7. Correlation Analysis: Tannins and Flavonoids vs. Biological Activity

The tannin or flavonoid contents of all tested methanol extracts were plotted against their biological activity (mean inhibition diameter of all tested bacteria or minimal active concentration (MAC), obtained in the biofilm assay, respectively) to check if there was any correlation. The data were analyzed by GraphPad Prism Version 6.07 [31]. The data were tested for normal distribution with Shapiro-Wilk test. Correlation was tested with Spearman Rank Correlation test, since the test showed to be more robust against outliers and violation of normal distribution assumption [29]. Correlation is assumed if correlation coefficient is significantly (*p* ≤ 0.05) non-zero (R_s_ > ±0.10) [32]. Correlation between direct antimicrobial activity and biofilm inhibition was tested in the same manner.

### 3.8. HPLC Fingerprints

For further analysis, HPLC fingerprints of the methanol extracts were recorded. Extracts were dissolved in methanol to a final concentration of 1 mg mL^−1^ (injection volume 20 µL). The following standard substances were used (each 0.1 mg mL^−1^, injection volume 5 µL): hyperoside (CarlRoth, D), quercetin (Sigma-Aldrich/Merck, D), isoquercitrin (CarlRoth, D), rosmaric acid (Sigma-Aldrich/Merck, D), (+)-catechin (CarlRoth, D), rutoside (Acros Organics/Thermo Scientific, Branchburg, NJ, USA), gallic acid (Acros Organics/Thermo Scientific, Waltham, MA, USA), umbelliferone (Sigma-Aldrich/Merck, D), and ellagic acid (Acros Organics/Thermo Scientific, Branchburg, NJ, USA). The HPLC configurations (Shimadzu LC20-AHT) were as follows: A Phenomenex^®^ Luna^®^ C18(2) (5 µm, 100 Å, 250 mm × 4.6 mm) was used as stationary phase and the column oven was set to 45 °C. Mobile Phase A was water with 0.1% acetic acid. Mobile phase B was acetonitrile with 0.1% acetic acid. The total flow was set at 1.2 mL min^−1^ with the following gradient: t_0min_ B14%, t_15min_ B16%, t_38min_ B80%, t_42.5min_ B80%, t_42.6min_ B14%, t_46min_ B14%. Detection was performed with UV detector at 254 nm.

## 4. Conclusions and Outlook

The presented study demonstrated that extracts of some traditionally used medicinal plants can affect the biofilm of multi-resistant gram-negative bacteria. Based on the data obtained from the correlation analysis, it can be deduced that tannins, but not flavonoids, might be responsible for this effect. To the best of our knowledge, for *Rubus chamaemorus* leaves, there are only few reports about phytochemical composition and biological activity. However, this is the first study reporting on anti-biofilm activity for *R. chamaemorus* leaves. The presented data suggest further investigation of the leaves of this plant, of which usually only the berries are used. *Aronia melanocarpa*, one of the novel “super foods”, showed no biological activity in this study. With HPLC fingerprints, it was possible to obtain an approximate overview of the components contained in the methanolic plant extracts.

Further studies will be conducted with those extracts that were most active. Of particular interest are *Epilobium angustifolium*, *Rubus chamaemorus,* and *Filipendula ulmaria*, as these proved to be particularly effective in the presented study. Bio-activity guided fractionation of the mentioned extracts and structural elucidation of active compounds will be the next steps. Furthermore, other strains from the WHO priority list will be included to check whether the principles of virulence reduction can also be applied to them.

## Figures and Tables

**Table 1:** Extract yield, and total tannin and flavonoid content (determined in plant biomass) of the tested medicinal plants.

Plant (Organ)	Extract Yield (%)			Content (% DW, Mean ± Range)		
	DCM	MeOH	Water	Tannins	Flavonoids (Boric Acid Method)	Flavonoid (AlCl_3_ Method)
*A. melanocarpa*^1^ (fruit)	4.89	65.14	3.85	3.37 ± 0.09	0.32 ± 0.06	0.09 ± 0.06
*A. uva-ursi*^2^ (leaves)	12.92	51.42	3.27	7.68 ± 0.67	1.20 ± 0.01	0.82 ± 0.01
*Cinchona* sp. ^2^ (bark)	0.35	19.83	2.66	2.15 ± 0.25	0.32 ± 0.10	0.03 ± 0.01
*C. paulstre*^1^ (herb)	8.10	20.13	7.20	5.26 ± 0.09	1.06 ± 0.21	0.31 ± 0.02
*C. palustre*^1^ (rhizome)	0.91	12.89	3.06	2.56 ± 0.19	0.09 ± 0.01	0.00 ± 0.02
*E. angustifolium*^1^ (leaves)	4.71	38.44	8.40	10.41 ± 0.78	1.92 ± 0.29	1.86 ± 0.11
*F. ulmaria*^1^ (herb)	3.00	47.63	7.74	7.57 ± 1.42	1.90 ± 0.00	1.28 ± 0.19
*G. rivale*^1^ (herb)	4.45	18.77	13.55	3.73 ± 0.48	0.60 ± 0.04	0.26 ± 0.04
*H. virginiana*^2^ (leaves)	2.62	19.84	3.79	5.30 ± 0.13	0.87 ± 0.00	1.59 ± 0.05
*K. lappacea*^2^ (root)	4.15	13.26	2.20	4.07 ± 0.28	0.14 ± 0.02	0.03 ± 0.00
*P. bistorta*^1^ (rhizome)	0.50	31.23	5.49	7.36 ± 0.80	0.02 ± 0.02	0.00 ± 0.00
*Quercus* sp. ^2^ (bark)	2.49	10.42	2.71	3.01 ± 0.05	0.10 ± 0.01	0.06 ± 0.01
*R. chamaemorus*^1^ (leaves)	5.50	29.04	10.07	8.96 ± 0.26	1.17 ± 0.45	0.37 ± 0.03
*S. officinalis*^1^ (root & rhizome)	0.85	28.42	7.48	8.26 ± 0.87	0.15 ± 0.05	0.04 ± 0.03

DCM = dichloromethane; MeOH = methanol; ^1^ = origin: Skt. Petersburg; ^2^ = origin: purchased from A. Galke GmbH, Bad Grund, Germany; *n* = 1 (extract yield) or 2 (tannins and flavonoids).

**Table 2:** Antimicrobial properties of plant extracts from tannin- and flavonoid-rich medicinal plants.

Extract	DCM							MeOH							Water						
Test Organism	*B. subtilis*	*S. aureus*	*E. coli*	*P. aeruginosa*	*C. maltosa*	*E. coli* PBio 729	*E. coli* PBio 730	*B. subtilis*	*S. aureus*	*E. coli*	*P. aeruginosa*	*C. maltosa*	*E. coli* PBio 729	*E. coli* PBio 730	*B. subtilis*	*S. aureus*	*E. coli*	*P. aeruginosa*	*C. maltosa*	*E. coli* PBio 729	*E. coli* PBio 730
Plant (Organ)	Inhibition Diameter (mm)							Inhibition Diameter (mm)							Inhibition Diameter (mm)						
*A. melanocarpa* (fruit)	-	7	-	-	-	-	-	-	-	-	-	-	-	-	-	-	-	-	-	-	-
*A. uva-ursi* (leaves)	9	-	-	-	9.5	-	-	8	11	8	-	8	-	-	7	-	8	-	-	-	-
*Cinchona* sp. (bark)	8	-	-	10	9	-	-	8	-	8	-	-	-	-	-	-	-	8	9,5	-	-
*C. palustre* (herb)	10	7	-	-	-	-	-	-	11	-	-	16	-	-	-	10	-	-	10	-	-
*C. palustre* (rhizome)	-	9	-	8	-	-	-	-	11	-	-	15	-	-	-	-	-	-	12	-	-
*E. angustifolium* (leaves)	-	-	-	-	-	-	-	-	10	13	12	25	-	-	-	14	11	-	18	-	-
*F. ulmaria* (herb)	-	14	-	-	-	-	-	-	10	-	-	17	-	-	-	-	-	-	9.5	-	-
*G. rivale* (herb)	-	-	-	-	-	-	-	-	9.5	9	-	19	-	-	-	-	-	-	12	-	-
*H. virginiana* (leaves)	-	-	9.5	-	-	-	-	8	12	10	12	8	-	-	-	8.5	8	-	-	-	-
*K. lappacea* (root)	11	11	8.5	-	9.5	-	-	9.5	9	8.5	-	14	-	-	-	-	-	-	-	-	-
*P. bistorta* (rhizome)	-	-	-	-	-	-	-	-	12	-	-	12	-	-	-	-	-	-	8	-	-
*Quercus* sp. (bark)	8	9	10	-	-	-	-	6.5	10	-	-	-	-	-	-	9	-	-	-	-	-
*R. chamaemorus* (leaves)	-	-	-	-	-	-	-	-	12	-	-	16	-	-	-	9.5	-	-	10	-	-
*S. officinalis* (root & rhizome)	-	-	-	-	-	-	-	-	12	8	-	18	-	-	10	9	-	-	16	-	-

Diameter of inhibition zone (in mm) for dichloromethane (DCM), methanol (MeOH), and water extracts. Test organisms: *Bacillus subtilis*, *Staphylococcus aureus*, *Escherichia coli*, *Pseudomonas aeruginosa*, *Candida maltosa*, *Escherichia coli* Strain PBio 730, *Escherichia coli* Strain PBio 729. Mean (*n* = 2). - = no activity.

**Table 3:** Anti-biofilm properties from tannin- and flavonoid-rich medicinal plants.

Extract	DCM		MeOH		Water	
Test Organism	*E. coli* PBio 729	*E. coli* PBio 730	*E. coli* PBio 729	*E. coli* PBio 730	*E. coli* PBio 729	*E. coli* PBio 730
Plant (Organ)	µg		µg		µg	
*A. melanocarpa* (fruit)	-	-	-	-	n.t.	n.t.
*A. uva-ursi* (leaves)	-	-	20	50	200	200
*Cinchona* sp. (bark)	-	-	-	200	-	-
*C. palustre* (herb)	-	-	30	50	100	100
*C. palustre* (rhizome)	-	-	50	80	200	200
*E. angustifolium* (leaves)	-	-	50	50	100	100
*F. ulmaria* (herb)	-	-	30	30	200	200
*G. rivale* (herb)	-	-	65	100	200	200
*H. virginiana* (leaves)	-	-	20	50	-	-
*K. lappacea* (root)	-	-	200	-	n.t.	n.t.
*P. bistorta* (rhizome)	-	-	10	10	-	-
*Quercus* sp. (bark)	-	-	30	50	100	100
*R. chamaemorus* (leaves)	-	-	20	50	100	100
*S. officinale* (root & rhizome)	-	-	20	40	200	200

Minimal active concentration (in µg mL^−1^) for dichloromethane (DCM), methanol (MeOH), and water extracts. Test organisms: *Escherichia coli* Strain PBio 730, *Escherichia coli* Strain PBio 729. Mean (*n* = 2). - = no activity; n.t. = not tested.

## Data Availability

Not applicable.

## Supplementary Materials

The following are available online at https://www.mdpi.com/article/10.3390/molecules27072284/s1, Table S1: Summarized statistical data for correlation analysis (Spearman Rank Correlation Test) of tannins or flavonoids vs. biological activity. Support for Figure 1 and Figure 2. Analysis performed with GrapPad prism Version 6.07, Table S2: Additional information how voucher specimen were identified. Support for Section 3.1.

## Sample Availability

Samples of the used plant material and of the used microorganisms are available from the authors.

## Abbreviations

DCM dichloromethane; MAC minimal active concentration; MeOH methanol; Ph.Eur. European Pharmacopoeia; RT retention time.
