# Supplementary material for: Evaluating Tannins and Flavonoids from Traditionally Used Medicinal Plants with Biofilm Inhibitory Effects against MRGN E. coli
Source: Molecules. 2022 Mar 31;27(7):2284. doi: 10.3390/molecules27072284 (PMC9000218; doi:10.3390/molecules27072284)
Supplement: Supplementary file 1 [file molecules-27-02284-s001.zip › molecules-1638828-supplementary.pdf]

Table S1. Summarized statistical data for correlation analysis (Spearman Rank Correlation Test) of tannins or flavonoids vs. biological activity. Support for figure 1 and 2. Analysis performed with GrapPad prism Version 6.07.

Compound	Tannins		Flavonoids ($AlCl_3$)		Flavonoids (Boric Acid)		
Correlation Value	Tannins (% DW) vs. Inhibition diameter	Tannins (% DW) vs. MAC (μg mL^{-1})	Flavonoids (% DW) vs. Inhibition Diameter	Flavonoids (% DW) vs. MAC (μg mL^{-1})	Flavonoids (% DW) vs. Inhibition Diameter	Flavonoids (% DW) vs. MAC (μg mL^{-1})	MAC vs. Inhibition Diameter
**Spearman coefficient (R_s)**	0.6205	−0.6131	0.5094	−0.2170	0.4251	−0.1082	0.01111
**95% Confidence interval**	0.1168 to 0.8703	−0.8745 to -0.07556	−0.04651 to 0.8244	−0.6955 to 0.3949	−0.1533 to 0.7866	−0.6332 to 0.4850	−0.5560 to 0.5712
**p value (two-tailed)**	0.0202	0.0288	0.0648	0.4716	0.1301	0.7242	0.9724
**Number of pairs**	14	13	14	13	14	13	13

Table S2. Additional information how voucher specimen were identified. Support for section 3.1.

№	Species	Nomenclatural Citation and Type Specimen Description (IPNI)	Image of the Type Herbarium Specimen (POWO)
1	*Aronia melanocarpa* (Michx.) Elliott	https://www.biodiversitylibrary.org/page/10166742#page/586/mode/1up (accessed on 16 August 2019)	http://apps.kew.org/herbcat/detailsQuery.do?barcode=K000758377 (accessed n 16 August 2019)
2	*Potentilla palustris* (L.) Scop.	https://www.biodiversitylibrary.org/page/58202898#page/481/mode/1up (accessed on 16 August 2019)	https://powo.science.kew.org/taxon/urn:lsid:ipni.org:names:63773-2 (accessed on 16 August 2019)
3	*Epilobium angustifolium* L.	https://www.biodiversitylibrary.org/page/358366#page/359/mode/1up (accessed on 16 August 2019)	http://apps.kew.org/herbcat/detailsQuery.do?barcode=K000914235 (accessed on 16 August 2019)
4	*Geum rivale* L.	https://www.biodiversitylibrary.org/page/358520#page/513/mode/1up (accessed on 16 August 2019)	https://powo.science.kew.org/taxon/725355-1 (accessed on 16 August 2019)
5	*Filipendula ulmaria* (L.) Maxim.	https://www.biodiversitylibrary.org/page/15733711#page/263/mode/1up (accessed on 16 August 2019)	http://apps.kew.org/herbcat/detailsQuery.do?barcode=K000914185 (accessed on 16 August 2019)
6	*Polygonum bistorta* L.	https://www.biodiversitylibrary.org/page/358379#page/372/mode/1up (accessed on 16 August 2019)	https://powo.science.kew.org/taxon/urn:lsid:ipni.org:names:60430545-2 (accessed on 16 August 2019)
7	*Rubus chamaemorus* L.	https://www.biodiversitylibrary.org/page/358513#page/506/mode/1up (accessed on 16 August 2019)	http://specimens.kew.org/herbarium/11665.000 (accessed on 16 August 2019)
8	*Sanguisorba officinalis*	https://www.biodiversitylibrary.org/page/358135#page/128/mode/1up (accessed on 16 August 2019)	http://specimens.kew.org/herbarium/K000914195 (accessed on 16 August 2019)
